# miR-21-5p Targets PIK3R1 to Regulate the NF-*κ*B Signaling Pathway, Inhibiting the Invasion and Progression of Prolactinoma

**DOI:** 10.1155/ije/7741091

**Published:** 2025-02-05

**Authors:** Min de Li, Juan Yang, Xiao Wu, Shang Si Chen

**Affiliations:** ^1^Department of Rehabilitation Medicine, Affiliated Rehabilitation Hospital of Nanchang University, Nanchang, Jiangxi 330006, China; ^2^Department of Neurosurgery, The First Affiliated Hospital of Nanchang University, Nanchang, Jiangxi 330006, China

**Keywords:** I*κ*Ba, invasion, migration, miR-21-5p, PIK3R1, prolactinoma

## Abstract

Prolactinomas (PRLs) are benign tumors with malignant characteristics that can invade the surrounding tissue structures and are challenging to treat. It has been reported that miR-21-5p expression in pituitary adenomas is correlated with tumor invasion and size. However, the mechanism of action of miR-21-5p in PRL remains unclear. Dysregulation of the phosphoinositide-3-kinase (PI3K) regulatory Subunit 1 pathway occurs frequently in cancer and plays an important role in tumor progression as an important component of the PI3K pathway. However, the role of PIK3R1 in PRL and its regulatory mechanism are unknown. In this study, we first explored the effect of miR-21-5p in PRL and then confirmed that PIK3R1 is a direct target of miR-21-5p using bioinformatics and cellular experiments. Subsequent in vitro experiments demonstrated that overexpression of PIK3R1 significantly attenuated the biological effects of miR-21-5p in PRL cells, such as promoting proliferation and invasion. Finally, we explored the mechanism by which PIK3R1 affects PRL progression and found that the inhibition of I*κ*Ba degradation by PIK3R1 impacts PRL progression via the miR-21-5p/PIK3R1/MMP pathway.

## 1. Introduction

Prolactinoma (PRL) is a tumor with endocrine function and is the most common tumor among all pituitary tumors [1]. Although PRL is a benign tumor, it has attracted much attention because of its distinct features, such as the anatomical location of the tumor and the symptoms produced by the tumor, including hyperprolactinemia that leads to amenorrhea, menstrual disorders, cessation of ovulation, male infertility, and sexual dysfunction in women. Some PRLs have invasive properties resulting in invasion of the surrounding tissues, which can lead to low vision, head pain, and can even be fatal [2]. Therefore, elucidating the pathogenesis of PRL is very important for its diagnosis and treatment. However, to date, studies on the molecular mechanism of PRL are very limited, and its molecular pathogenesis is controversial. The prevailing theory is that pituitary tumors are monoclonal tumors and that their proliferation stems from mutations in individual pituitary cells. However, it is unclear which pituitary cell mutations are more important for the local regulation of growth factors in the hypothalamus. Therefore, further research on the molecular mechanism of PRL is urgently needed.

miR-21 is overexpressed in many tumors, and reports have showed that miRNA-21 can play an important role in tumor proliferation and invasion in Type B malignant lymphoma, breast cancer, pancreatic cancer, and glioma [3–6]. Recently, studies have indicated that the interaction between miR-21-5p and long noncoding RNAs (lncRNAs) plays a significant role in the invasion and progression of various types of cancer [7]. It has also been reported that miR-21 expression is closely related to biological behaviors such as invasion and proliferation of pituitary adenomas [8, 9]. However, there is a lack of research on the role of miR-21 (miR-21-5p) in PRLs. In this study, we investigated the effect of miR-21 (miR-21-5p) on PRL at the cellular and animal levels.

As a major regulator of human cancer, phosphatidylinositol 3-kinase (PI3K) is a major signaling component downstream of growth factor receptor tyrosine kinase (RTK) [10]. In PRL, the peripheral prolactin receptor (PRLR), a member of the cytokine receptor superfamily, has been reported to transduce signals through the phosphoinositide 3-kinase-Akt (PI3K-Akt) or MAPK pathways to mediate changes in transcription, differentiation, and proliferation [11]. Numerous drugs tested in PRL cell lines have shown to exert inhibitory effects on tumor proliferation, mainly through the PI3K/AKT pathway [12]. Moreover, somatic mutations of the components involved in the activation of the PI3K pathway include mainly PIK3CA encoding the PI3K catalytic subunit p110*α*, PIK3R1 encoding the PI3K regulatory subunit p85*α*, and other genes encoding PI3K-related regulators, and it has been reported that PIK3R1 encodes p85a, followed by the action of downstream MMPs to play a tumor suppressor role [13–16]. The main mechanism underlying this effect is that after activation by PI3K/AKT, the transcription factor NF-*κ*B is separated from I*κ*B by the activation of IKK (I*κ*B kinase), which phosphorylates I*κ*Ba and translocates to the nucleus to induce the expression of MMPs [17–19]. Therefore, based on previous studies, it is clear that it is additional studies on the mechanism of action of PIK3R1/MMPs in RPL are needed.

In this study, we selected MMP12 and MMP14 as downstream target genes of PIK3R1 to investigate whether PIK3R1 in PRL affects the progression of PRL through miR-21-5p/PIK3R1/MMPs.

## 2. Materials and Methods

Rat pituitary tumor cell line (Procell) contain the following: GH3, CL-0590 and MMQ, CL-0340.

### 2.1. PRL Samples

#### 2.1.1. Patient Selection Criteria

The patients are selected based on the following criteria: (1) patients diagnosed with PRL; (2) patients who did not receive any radiotherapy, chemotherapy, or other related treatments before operation; (3) all patients had no other tumors except PRL; (4) patients with other underlying diseases, such as diabetes, hypertension, or severe heart disease, were excluded; (5) other family history of genetic diseases was negative. The relevant parameters of the enrolled patients are shown in Table 1.

### 2.2. Tissue Samples

**Figure 1:**
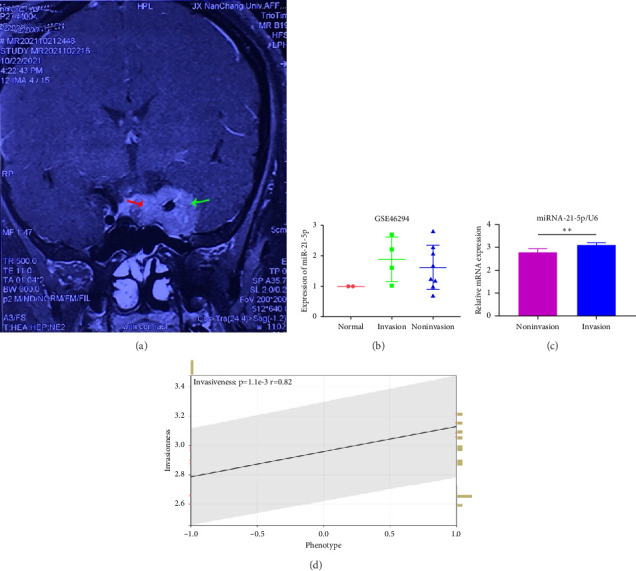
miR-21-5p expression is significantly increased in invasive PRLs, and miR-21-5p expression is positively correlated with invasiveness. (a) Magnetic resonance imaging (MRI) and sampling sites of the included PRL patients; red arrows indicate juxtapituitary regions, weakly invasive regions, or none; green arrows indicate distal pituitary tumors and strongly invasive regions; (b) GSE46294 data analysis revealed that miR-21-5p expression was significantly greater in invasive PRL than in noninvasive PRL. (c) PCR detection of clinical samples revealed that the expression of miR-21-5 in invasive pituitary tumors was significantly greater than that in noninvasive tissues (*p* < 0.001). (d) Spearman correlation analysis revealed that the expression of miR-21-5p was positively correlated with invasiveness (*R* = 0.82, *p*=0.0011). The data in C are representative of 3 independent experiments and are presented as the means ± SDs; ⁣^∗^*p* < 0.05 and ⁣^∗∗^*p* < 0.01.

Twelve pairs of fresh PRL specimens were obtained after surgery from patients diagnosed with PRL (serum prolactin levels > 470 ng/mL) admitted to the First Affiliated Hospital of Nanchang University from January 2018 to October 2021. Each pair included two parts of tissue: intrasellar—juxtapituitary and cavernous sinus—away from the pituitary (away from the normal pituitary), which we considered to be more invasive. We isolated invasive PRLs (invasion part, Figure 1(a), green arrow) near pituitary tumor tissue (normal, Figure 1(a), red arrow), as tumors break through their capsule and invade the cavernous sinus farther away. The obtained tissues were stored immediately in a −80°C freezer. All enrolled patients were diagnosed with PRL according to the Chinese Consensus for the Diagnosis and Treatment of Pituitary PRL (2014 Edition) (specific diagnostic indicators had typical clinical manifestations, hyperprolactinemia, and imaging examination) [92]. Moreover, the case data of all patients were accurately and carefully recorded. This study was approved by the Ethics Committee of the First Affiliated Hospital of Nanchang University. Informed consent was obtained from the patients for the operation and tissue specimen collection, and relevant informed consent forms were signed before the operation.

### 2.3. Cell Culture and Subculture

For cell culture, MMQ and GH3 cells (Procell CL-0590) were cultured in Ham's F-12K + 15% HS + 2.5% FBS + 1% medium (penicillin streptomycin solution) in a humidified incubator at 37°C and 5% CO_2_.

### 2.4. Cell Transfection

GH3 and MMQ cells in the logarithmic growth phase were inoculated in a 6-well plate at 1 × 10^5^ cells/well and cultured overnight at 37°C and 5% CO_2_ saturated humidity. Two hours before transfection (CDNA, Youbao Biology, Hunan, China), the serum-free medium was used. The transfected cells were divided into the following groups: (1) normal cells: NC, (2) normal cells: NC mimics, and (3) miR-21-5p overexpression plasmid: miR-21-5p mimics. Finally, the cells were placed in an incubator at 37°C and a concentration of 5% CO_2_. After 6 h of culture, the medium was changed to normal medium and incubated for 24 h at 37°C with 5% CO_2_.

### 2.5. Transwell Assay

The collected cells, including those from the normal group and the transfection group, were resuspended, counted, diluted, and added to prepared 24-well plates. The transwell chamber was then placed on top of the plate. One hundred microliters of 1 mg/mL Matrigel matrix gel was added vertically to the center of the bottom of the upper chamber. After incubation, the target cells were inoculated, and the chamber was incubated again. Following incubation, the cells were fixed and stained. Finally, the invasive and migratory cells were observed and photographed under a microscope.

### 2.6. Real-Time Quantitative PCR

TRIzol reagent (Life Technologies) was used for RNA extraction. The synthesis of complementary DNA (cDNA) was performed with a Prime Script RT kit (Takara) and the first strand of Mir-X miRNA (Takara). The other procedures were the same as those in Part I. The miR-21-5p sequence was as follows: hsa miR-21-5p loop primer GTCGTATCCAGTGCAGG GT CCGAGGTATCGCA CTGGATCCAACCTC; F primer TGCGCTAGCTTA TCA GACTGA. The U6 and GAPDH primers were synthesized by Novozan Biotechnology Co., Ltd. qRT–PCR was used to determine the transcription level of some genes.

### 2.7. CCK-8

A CCK-8 kit (Sigma) was used to assess cell viability. The transfected GH3 and MMQ cells were seeded onto a 96-well plate at a density of 2 × 10^4^ cells per well. Ten microliters (10 μL) of CCK-8 assay reagent was added to each well, and the plate was incubated for 2–4 h. The optical density (OD) values at 450 nm were subsequently measured using a microplate spectrophotometer for each well.

### 2.8. TUNEL Staining

The treated cells (GH3 slime, MMQ smear) were fixed in 4% paraformaldehyde (pH 7.4) solution at room temperature for 15 min. After being washed with PBS, centrifuged, and resuspended, the cells were incubated at room temperature for 15 min. The cells were incubated with bright red labeling mix on ice. The prepared TdT (Vazyme, A113-03) incubation buffer was added to the slides, and the slides were incubated for 60 min at 37°C (wrapped in aluminum foil paper in the dark). Finally, the core was restained and an image was acquired.

### 2.9. Tumorigenesis Assay

#### 2.9.1. Animal Models

Firstly, nude mice were subjected to adaptive feeding for 7 days. For inoculation, 200 μL of cell suspension (containing approximately 1 × 10^7^ cells) was injected after transfection and other treatments in the right axilla of nude mice, after wiping and disinfecting with alcohol cotton balls, and pinhole hemostasis was performed to continue cage feeding. The survival status and tumor size of the nude mice were observed and recorded, and after tumor emergence, the nude mice were weighed and recorded twice a week. The length and diameter of the tumors were measured with a Vernier caliper and recorded as *a* and *b*, and the tumor volume calculation formula V = ab2/2 was used.

When the tumor volume reached 60 mm^3^, the nude mice were randomly divided into two groups, with three nude mice in each group: (1) the normal group and (2) the miR-21-5p overexpression group.

On the 33rd day of the experiment, all the nude mice were sacrificed via cervical dislocation. The tumors were removed, neatly placed in a monochromatic background according to groups, and photographed with a ruler. Then, half of the tumor tissues were fixed in 4% paraformaldehyde and half were cryopreserved for relevant index detection.

### 2.10. Immunohistochemistry

The tissue blocks were analyzed using standard immunohistochemical experimental procedures for gradient alcohol for tissue dehydration, tissue transparency, redehydration, wax immersion, embedding, sectioning and baking, dewaxing, antigen retrieval, blocking endogenous peroxidase, serum blocking, spiking primary antibody, spiking secondary antibody, spiking chromogen counterstaining, dehydration, mounting, and microscopic photography (not repeated), and finally, OD analysis of immunohistochemical photographs was performed using IPP6.0 software. The dilution concentration of primary antibody against Ki67 was 1:100.

### 2.11. Luciferase Reporting Assay

The groups were as follows: (1) normal 293T control cells, (2) WT-PIK3R1 3′UTR reporter plasmid + NC mimics, (3) WT-PIK3R1 3′UTR reporter plasmid + miR-21-5p mimics, (4) MUT-PIK3R13′UTR reporter plasmid + NC mimics, and (5) MUT-PIK3R1 3′UTR reporter plasmid + miR-21-5p mimics. After the cultured cells were exhausted, 200 μL of reporter cell lysate was added. After full lysis and centrifugation at 12,000 RPM for 3 min, the supernatant was collected for analysis. The firefly luciferase detection reagent and Renilla luciferase detection buffer were dissolved and incubated at room temperature. Renilla luciferase detection substrate (100×) was placed in an ice bath or an ice box for use. An appropriate amount of Renilla luciferase detection buffer was added to each sample (100 μL), and Renilla luciferase detection substrate (100×) was added at a ratio of 1:100 to prepare a Renilla luciferase detection working solution. Fifty microliters of sample was added, 100 μL of firefly luciferase detection reagent was added, the mixture was mixed well, and the RLU was determined. One hundred microliters of Renilla luciferase was added to the working solution, and the RLU was determined after mixing. With firefly luciferase as an internal reference, the RLU value determined by Renilla luciferase was divided by the RLU value determined by firefly luciferase.

### 2.12. Western Blot Analysis

The cells were collected on ice, lysed with lysis buffer, and centrifuged at 12, 000 × *g* for 30 min, after which the supernatant was retained. Protein lysates were loaded onto SDS-GEL, separated via SDS–PAGE and transferred to PVDF membranes. After blocking with 20% (PBS-Tween) and 5% BSA for 1 h, the film was incubated overnight with the following primary antibodies: anti-GAPDH (Hangzhou Xian Zhi Biology Co., Ltd., AB-P-R001), rabbit IKB-a (39 kDa Affinity AF5002), rabbit p-IKB-a (39 kDa Affinity AF3239), rabbit PI3K (85 kDa Affinity AF6241), rabbit MMP14 66 kDa AF0212);, and rabbit MMP12 (54 kDa DF7686), followed by washing in Tris-buffered saline with Tween 20 (10 min, 3 washes). Then, the sections were incubated with the appropriate secondary antibody (1:5000; Wuhan Baishi Biological Engineering Co., Ltd., BA1054). The signal was observed using enhanced chemiluminescence reagent (Tianjin Hanzhong Photographic Materials Factory), and density analysis was performed using ImageJ (National Institutes of Health).

### 2.13. Principal Component Analysis (PCA) of the Microarray Data

PCA was performed using R package stats (version 3.6.0) for GSE32191, which was standardized to determine the reproducibility of the data.

### 2.14. Heatmap, Volcano Plot, and Differential Expression Gene Analysis

Heatmaps and volcano plots were generated using the R package “heatmap” with “ggplot2.” Analysis of differentially expressed genes (DEGs) was performed on the dataset using the R package “limma.” Genes with *p* < 0.05 were considered differentially expressed.

### 2.15. Venn Diagrams

The online website Draw Venn Diagrams (https://bioinformatics.psb.ugent.be/webtools/venn/) was used for Venn diagrams.

### 2.16. Protein-Protein Interaction (PPI) Network Diagram Analysis

The STRING database was used to draw PPIs. Cytoscape software (version 3.9.1) and cytoHubba were used to analyze the hub genes.

### 2.17. miRNA Prediction Platform for Prediction of Target Genes for miR-21-5p

The online web target prediction platforms used included miRTarBase (miRTarBase [Mayanlab.cloud]), miRWalk (Home-miRWalk [Fig. uni-heidelberg.de]), miRDB (miRDB-MicroRNA Target Prediction Database), and TargetScan (TargetScanHuman 7.1). The TargetScan platform was used to predict data for two species, humans and mice, and a total of five sets of data were obtained. The GEO database GSE32191 microarray data were downloaded (the annotation platform of GSE32191 is GPL2895), which contained 8 invasive samples and 5 noninvasive samples. DEGs were analyzed between the invasive and noninvasive groups.

### 2.18. Statistical Analysis

Statistical analysis was performed using SPSS software (version 20.0; IBM Company). Correlation analysis was performed via the Spearman correlation. Student's *t* test and one-way analysis of variance were used to calculate differences between 2 or more groups. *p* < 0.05 was considered statistically significant (*p* < 0.05, *p* < 0.01, and *p* < 0.001; ns = not significant).

## 3. Results

### 3.1. miR-21-5p Expression Is Significantly Higher in PRL Tissues Than in Normal Tissues, and Its Expression Is Positively Correlated With Invasiveness

In this study, we performed preoperative magnetic resonance imaging (MRI) on the included patients. All samples were selected and labeled as shown in Figures 1(a): red arrows indicate the juxtapituitary regions, areas of weak invasiveness, or no invasiveness; green arrows indicate the distal pituitary tumors and areas of strong invasiveness. The relevant parameters of the included patients are shown in Table 1.

GEO2R analysis of miR-21-5p differential expression in invasive versus noninvasive pituitary tumors in the GSE46294 dataset revealed that miR-21-5p expression was significantly higher in invasive PRLs than in noninvasive PRLs (Figures 1(b)). A PCR assay of clinical samples revealed (Figures 1(c)) that miR-21-5p expression in invasive PRL tissues invading the cavernous sinus was significantly higher than that in noninvasive tissues, and the results were consistent with the GEO data. To elucidate the correlation between miR-21-5p and PRL invasiveness, we defined invasive PRL (Y value set as 1) in 4 of the 12 samples in which the tumor broke through its capsule and invaded the cavernous sinus and noninvasive PRL (*Y* value set as −1) in the other 8 samples in which the PRL did not invade the cavernous sinus, and the correlation between the expression of miR-21-5p, and the invasive phenotype was calculated using the Spearman correlation statistics. The results revealed that the expression of miR-21-5p was positively correlated with PRL invasiveness, with a correlation of *R* = 0.82 and *p*=0.0011, as shown in Figure 1(d).

### 3.2. miR-21-5p Promotes the Progression of PRL In Vitro and In Vivo

**Figure 2:**
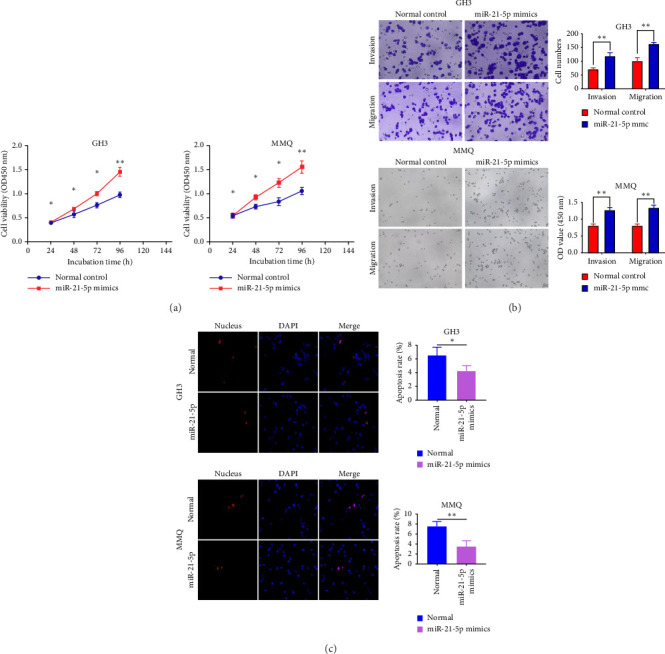
(a) miR-21-5p promotes PRL cell line proliferation (GH3, MMQ). miR-21-5p overexpression significantly enhanced PRL cell proliferation. (b) miR-21-5p promoted PRL cell invasion and migration. The invasion and migration of PRL cell lines (GH3, MMQ) were significantly increased after overexpression of miR-21-5p; (c) miR-21-5 inhibits PRL cells apoptosis. After the overexpression of miR-21-5p, the number of apoptotic PRL cell lines (GH3, MMQ) was significantly reduced (the data are representative of 3 independent experiments and are presented as the means ± SDs; ⁣^∗^*p* < 0.05 and ⁣^∗∗^*p* < 0.01).

**Figure 3:**
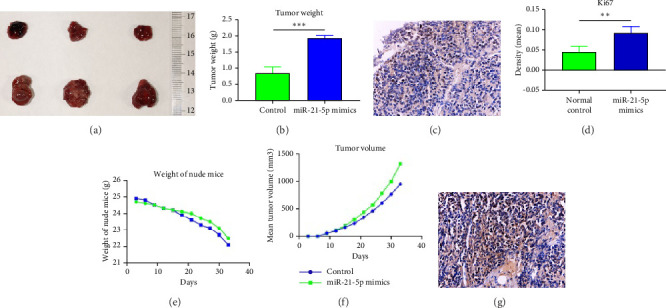
The effects of miR-21-5p in PRL in vivo include promoting tumor proliferation and invasion. (a, b) The tumor weight in the miR-21-5p mimic group was significantly greater than that in the control group; (c, g, d) Ki67 values were significantly greater in the miR-21-5p mimic group than in the control group; (e) there was no significant difference in body weight between the miR-21-5p mimic group and the control group during the duration of the experiment, indicating that there was no significant difference in the health status of the mice between the two groups; (f) tumor volume was significantly greater in the miR-21-5p mimic group than in the control group. (The data are representative of 3 independent experiments and are presented as the means ± SDs; ⁣^∗^*p* < 0.05 and ⁣^∗∗^*p* < 0.01).

To investigate the effect of miR-21-5p on the biological behavior of PRL cells, we overexpressed miR-21-5p in PRL cells and detected the effect of overexpressing miR-21-5p on the biological behavior of PRL cells using CCK8, transwell, and TUNEL assays. The results revealed that the proliferation (as shown in Figure 2(a)), invasion, and migration (as shown in Figure 2(b)) of PRL cells were significantly increased, and that apoptosis was attenuated after miR-21-5p overexpression (as shown in Figure 2(c)). PRL cells can promote PRL cell proliferation, invasion, and migration and inhibit apoptosis. Moreover, miR-21-5p promoted the proliferation and invasion of PRL tumors in vivo (as shown in Figure 3).

### 3.3. PIK3R1 Is a Hub Gene for PRL Invasiveness

**Figure 4:**
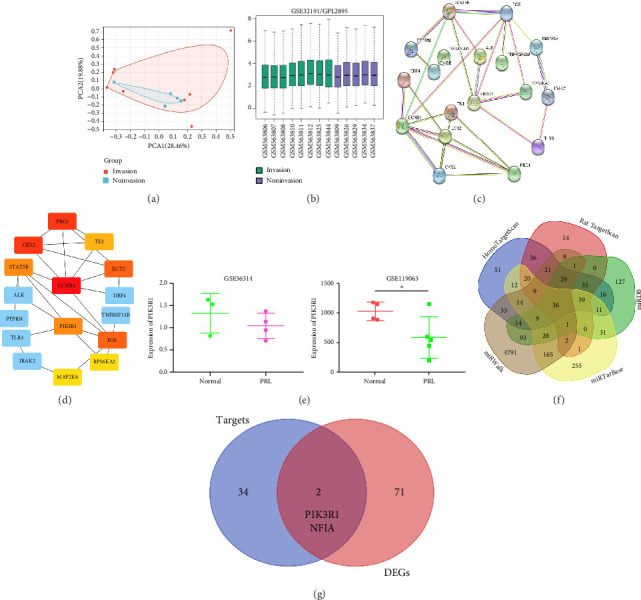
PIK3R1 is a hub gene for PRL invasion and progression. (a, b) are the principal component analysis results of GSE32191 and the boxplots after normalization, respectively, and the results show that the GSE32191 data have good reproducibility. (c) GSE32191 DEG (top 50) PPI plot and (d) cytoHubba analysis result. These results revealed that PIK3R1 is a hub gene for PRL invasiveness. (e) GEO dataset (GSE36314 and GSE119063) analysis revealed higher expression of PIK3R1 in PRLs than in the normal pituitary. (f) Target prediction results revealed that PIK3R1 was a direct target of miR-21-5p. (g) Predicted target genes and DEG Venn diagram. The results revealed that PIK3R1 is the target gene (⁣^∗^ indicates *p* < 0.05, ⁣^∗^ indicates *p* < 0.01).

To further explore the downstream mechanism of miR-21-5p, we first analyzed GSE32191 to obtain the hub gene set of PRL invasiveness (Figures 4(a) and 4(b) PCA and box plots for GSE32191, respectively), and these results demonstrated the good reproducibility of the GSE32191 data. GSE32191 was used for differential gene expression (DEG) analysis, and the top 50 DEGs were used for PPI network analysis (Figure 4(c)), followed by network mapping of the top 50 DEGs using cytoHubba interactions (Figure 4(d)). These data indicate that PIK3R1 is the s-hub gene for PRL invasiveness. The GSE36314 and GSE119603 datasets subsequently verified that PIK3R1 expression was significantly higher in PRL patients than in normal controls, as shown in Figure 4(e). We used the online miRNA target prediction platforms miRTarBase miRWalk, miRDB, and TargetScan to predict the set of target genes for miR-21-5p, as shown in Figure 4(f). Subsequently, a Venn diagram of the predicted target genes against the differential genes of GSE32191 revealed that PIK3R1 and NFIA were the target genes, as shown in Figure 4(g). These results indicate that PIK3R1 is a key gene for PRL invasiveness.

### 3.4. PIK3R1 Is a Direct Target of miR-21-5p and Inhibits PRL Proliferation

**Figure 5:**
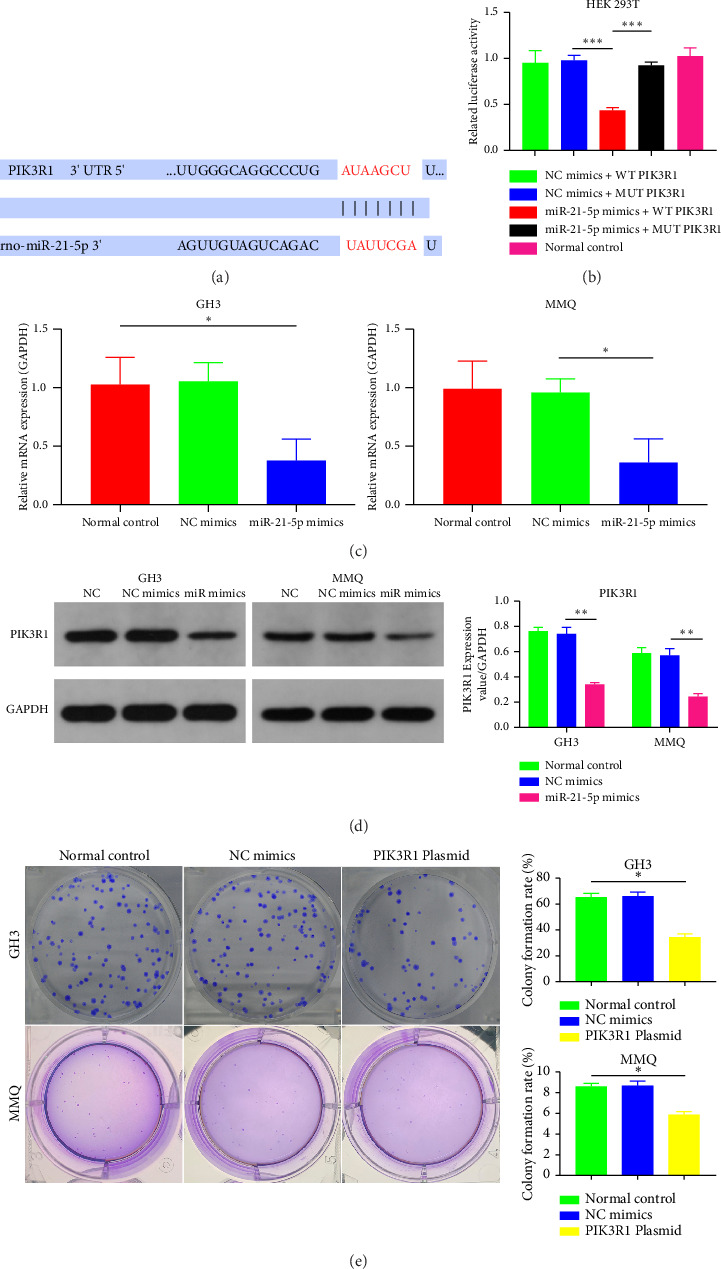
PIK3R1 is a direct target of miR-21-5p and inhibits PRL proliferation. (a) The paired sequence of miR21-5p and the 3′UTR of PIK3R1; (b) the dual-luciferase assay revealed that PIK3R1 was a direct target of miR-21-5p. (c) miR-21-5p mimics inhibited the expression of PIK3R1 mRNA in GH3 and MMQ cells. (d) miR-21-5p decreased the protein expression of PIK3R1 in GH3 and MMQ cells. (e) Cloning assays revealed that PIK3R1 significantly inhibited the proliferation and progression of PRL cells, and the results revealed that the colony formation rate in the PIK3R1 plasmid group was significantly lower than that in the other groups. (The data in (b–e) are representative of 3 independent experiments and are presented as the means ± SDs; ⁣^∗^*p* < 0.05, ⁣^∗∗^*p* < 0.01, and ⁣^∗∗∗^*p* < 0.001).

Recently, a report indicates that miR-21 targets PIK3R1 to influence the progression of various tumors. However, there are currently no reports on its role in PRL [20]. We used the prediction platform TargetScan to predict base complementary pairing of miR-21-5p with the 3′UTR of PIK3R1, as shown in Figure 5(a). Then, dual-luciferase assays were performed to further validate whether PIK3R1 is a target of miR-21-5p, and dual-luciferase reporter assays were performed after cotransfection of miR-5p 21-mimics and WT-PIK3R1 3′UTR/MUT-PIK3R1 3′ UTR in 293T cells. The results revealed that the luciferase activity of the PIK3R1-3′UTR wild-type reporter plasmid was significantly inhibited after transfection with the miR-21-5p mimics, but there was no significant change in the luciferase activity of the PIK3R1-3′UTR mutant reporter plasmid (Figure 5(b)). Thus, these results indicate that PIK3R1 is a direct target gene of miR-21-5p. Subsequent cell experiments revealed that the overexpression of miR-21-5p in PRL cells inhibited PIK3R1 expression at both the mRNA and protein levels, as shown in Figures 5(c) and 5(d). Cell cloning assays revealed that PIK3R1 significantly inhibited the proliferation and progression of PRL cells (Figure 5(e)).

### 3.5. PIK3R1 Inhibits I*κ*Ba Degradation, Affecting the Progression of PRL via the miR-21-5p/PIK3R1/MMP Pathway

**Figure 6:**
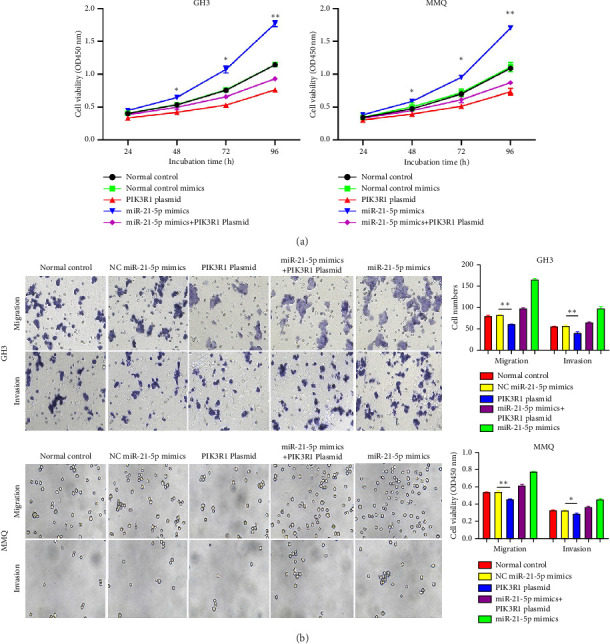
(a) CCK8 assay of GH3 and MMQ cells revealed that the overexpression of PIK3R1 significantly inhibited the ability of miR-21-5p to promote the proliferation of PRL cells. (b) Transwell assays of GH3 and MMQ cells revealed that overexpression of PIK3R1 significantly reduced the promoting effect of miR-21-5p on cell migration and invasion. The data are representative of 3 independent experiments and are presented as the means ± SDs; ⁣^∗^*p* < 0.05, ⁣^∗∗^*p* < 0.01, and ⁣^∗∗∗^*p* < 0.001.

**Figure 7:**
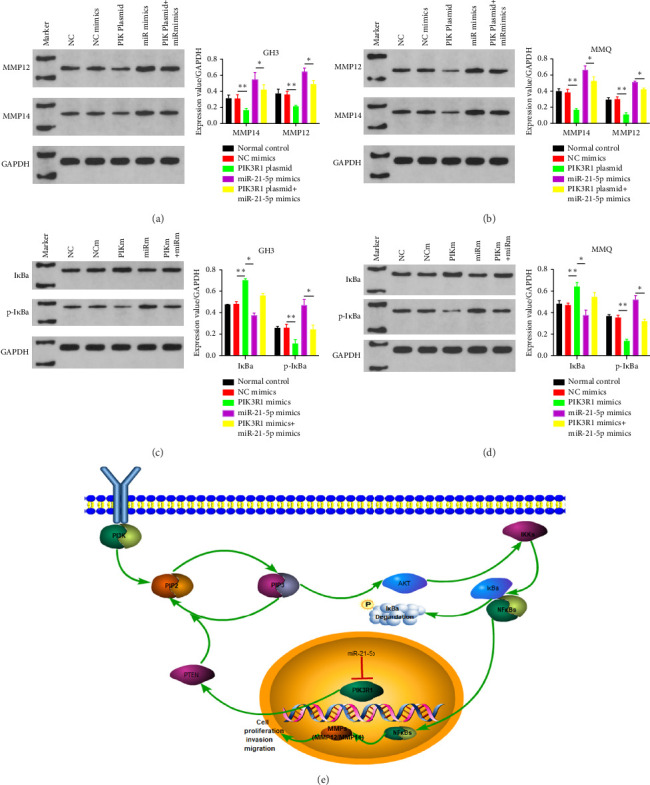
PIK3R1 inhibits I*κ*Ba phosphorylation and reduces I*κ*Ba degradation and MMP12/MMP14 in MMQ and GH3 cells. The results revealed that the overexpression of PIK3R1 decreased I*κ*Ba phosphorylation and increased total I*κ*Ba. PIK3R1 inhibits I*κ*Ba phosphorylation and decreases MMP12/MMP14 expression through miR-21-5p-PIK3R1-I*κ*Ba pathway in MMQ and GH3 cells (as shown in a–d). PIK3R1 inhibits I*κ*Ba degradation, thereby affecting the progression of prolactinoma via the miR-21-5p/PIK3R1/MMPs pathway (as shown in (e)). (The data are representative of 3 independent experiments and are presented as the means ± SDs; ⁣^∗^*p* < 0.05, ⁣^∗∗^*p* < 0.01, and ⁣^∗∗∗^*p* < 0.001).

On the basis of the previous experimental results, we transfected miR-21-5p mimics and PIK3R1 plasmids into GH3 and MMQ cells and then performed CCK8 and transwell assays to determine whether overexpression of PIK3R1 can reduce the ability of miR-21-5p to promote the proliferation, invasion, and migration of PRL-producing cells. CCK8 and transwell assays revealed that the proliferation, invasion, and migration abilities of the cells in the miR-21-5p mimics + PIK3R1 plasmid group were significantly lower than those in the miR-21-5p mimics + empty transfer group, and there was no significant difference between the two groups and the NC mimics + empty transfer group. As shown in Figures 6(a) and 6(b), these results demonstrated that the overexpression of PIK3R1 can inhibit the promoting effect of miR-21-5p in PRL cell proliferation, invasion, and migration. As described above, we transfected miR-21-5p mimics and PIK3R1 plasmids into GH3 and MMQ cells and then examined whether overexpression of PIK3R1 could inhibit the effects of miR-21-5p intervention on genes related to PIK3R1 downstream genes in PRL cells. The results revealed that I*κ*Ba phosphorylation was significantly lower and that the total amount of I*κ*Ba was significantly greater in the PIK3R1 plasmid group than in the other groups (as shown in Figures 7(a) and 7(b)). Moreover, the expression of MMP12/MMP14 was significantly lower in the PIK3R1 plasmid group than in the other groups (as shown in Figures 7(c) and 7(d)). PIK3R1 inhibits I*κ*Ba degradation, thereby affecting the progression of PRL via the miR-21-5p/PIK3R1/MMPs pathway (as shown in Figure 7(e)).

## 4. Discussion

At present, the treatment of PRL remains controversial, and consensus guidelines recommend dopamine agonists (DAs) as first-line treatments [21, 22], and surgery is indicated in patients who are resistant to DAs or cannot tolerate the side effects, or in cases of cystic fibroadenoma and persistent tumor bleeding with visual impairment [23–27]. In 2006, the Pituitary Society revised the guidelines, stating that surgery can be used as a first-line treatment with respect to patients' wishes (some patients cannot receive long-term DAs, even if they do not experience resistance or intolerance) [28]. However, there are still several limitations and risks in surgical treatment, such as whether the tumor can be completely removed by surgery and the possible risks and complications during and after surgery. Therefore, further studies on the mechanism of PRL occurrence and progression are necessary.

miR-21-5p has been reported to play an important role in the development and progression of a variety of tumors and diseases, including gastric cancer, lung cancer, rectal cancer, glioma, and melanoma [29–33]. Tang et al. reported that miR-21-5p plays an important role in the progression of lung cancer [34]. Zhang et al. reported that miR-21-5p plays an important role in the proliferation and progression of acute myeloid leukemia [35]. Li et al. reported that miR-21-5p plays an indispensable role in the progression of osteosarcoma [36], and it has also been shown that miR-21 expression in pituitary adenomas is closely related to tumor invasion and size [37]. However, whether miR-21-5p promotes or inhibits PRL biobehavior has not previously been reported.

In this study, both the GEO database data and the PCR results of the clinical samples revealed that the expression of miR-21-5p in the invasive part of PRL was significantly greater than that in the normal and noninvasive parts. In addition, the expression level of miR-21-5p was positively correlated with the invasiveness of PRL, and the more invasive the PRL was, the higher the expression level of miR-21-5p was.

The in vitro data revealed that miR-21-5p promoted the proliferation, migration and invasion of PRL cells and reduced their degree of apoptosis. In vivo experiments also demonstrated that miR-21-5p significantly promoted tumor growth in vivo without significant differences in body weight in nude mice (indicating that nude mice are similar under physiological conditions). Related data on Ki67 also suggest that miR-21-5p is able to promote PRL invasiveness [38, 39]. To identify the target of miR-21-5p in PRL, we first searched for potential target gene sets through a target prediction platform, and DEG analysis was subsequently performed on the GSE32191 invasive and noninvasive samples to identify DEGs. PIK3R1 was ultimately identified as the target gene. Dual-luciferase assays revealed that PIK3R1 is a direct target of miR-21-5p, which can directly act on PIK3R1 and inhibit the expression of PIK3R1 mRNA and protein. Cloning experiments further demonstrated that PIK3R1 can significantly inhibit the proliferation and progression of PRL cells.

Dysregulation of the PI3K pathway is well known to play an important role in the progression of numerous tumors including PRL [40, 41]. The role of PIK3R1, one of the major components of somatic mutations in the PI3K pathway, in the PI3K pathway is critical [42, 43]. Therefore, it is necessary to study the role of PIK3R1 in PRL and its related mechanisms.

PIK3R1 encodes P85a, which stabilizes PTEN, and PTEN induces the dephosphorylation of PIP3 to PIP2, which plays a role in tumor suppression [15, 44]. PI3K recruits PDK1 and AKT proteins to the plasma membrane, causing PDK1 to phosphorylate threonine 308 (T308) at position 308 of the AKT protein, resulting in partial activation of AKT, whereas at rest, I*κ*Ba binds to two subunits of NF*κ*B (p6 and, p50) in an inactivated state and is present in the cytoplasm. When upstream genes activate AKT, AKT activates IKK, and activated IKK undergoes ubiquitination and phosphorylation to degrade I*κ*B*α*, ultimately activating the two subunits of NF-*κ*B and further transferring them from the cytoplasm into the nucleus to promote the expression of MMPs, which in turn promotes enhanced biological behaviors such as cell proliferation, invasion, and migration [16, 45, 46].

In this study, we found that PIK3R1 overexpression promoted decreased I*κ*Ba phosphorylation and increased I*κ*Ba; then, I*κ*Ba bound to more NF*κ*B subunits to inactivate NF*κ*B, and the cancer-promoting effect of NF*κ*B was significantly reduced, which is consistent with the findings of previous studies. Thus, our data demonstrate that PIK3R1 induces reduced expression of MMPs (MMP12/MMP14) by reducing I*κ*Ba phosphorylation and ultimately leads to diminished proliferation, invasion, and migration of PRL cells.

In summary, the mechanism of the inhibitory effect of PIK3R1 in PRL is as follows: PIK3R can stabilize PTEN, followed by PTEN, to increase PIP3-to-PIP2 conversion, and attenuate the effect of the PI3K-PIP2-PIP3-AKT pathway [15, 44, 47]; subsequently, I*κ*Ba phosphorylation is reduced, I*κ*Ba content is increased (I*κ*Ba will bind to more NF*κ*B subunits to inactivate NF*κ*B), and the cancer-promoting effect of NF*κ*B is significantly reduced [16, 45, 46]), resulting in reduced expression of MMPs (MMP12/MMP14) to achieve a tumor suppressor effect. In conclusion, our data demonstrate that PIK3R1 reduces I*κ*Ba phosphorylation and impacts PRL progression via the miR-21-5p/PIK3R1/MMP pathway (Figure 7).

In summary, the interaction of miR21-5p with PIK3R1 triggers I*κ*Ba phosphorylation, which is reduced by PIK3R1 and affects the progression of PRL via the miR-21-5p/PIK3R1/MMP pathway. This pathway is therefore an important potential target to prevent and treat PRL.

## Figures and Tables

**Table 1:** Patient parameters.

Parameters	Tumor invaded into the cavernous	
	Yes	No
Sex		
M	0	5
F	3	4
Age (year)		
10–20	3	0
> 20	1	8

## Data Availability

The datasets generated and analyzed during the current study are available in the GEO Repository (https://www.ncbi.nlm.nih.gov/geo/query/acc.cgi?acc=GSE46294, https://www.ncbi.nlm.nih.gov/geo/query/acc.cgi?acc=GSE32191, https://www.ncbi.nlm.nih.gov/geo/query/acc.cgi?acc=GSE36314, and https://www.ncbi.nlm.nih.gov/geo/query/acc.cgi?acc=GSE119063). For additional data, please contact the corresponding author (email: liminde@email.ncu.edu.cn).

## References

[1] Daly A. F., Rixhon M., Adam C., Dempegioti A., Tichomirowa M. A., Beckers A. (2006). High Prevalence of Pituitary Adenomas: a Cross-Sectional Study in the Province of Liege, Belgium. *The Journal of Clinical Endocrinology & Metabolism Endocrinol Metab*.

[2] Klibanski A. (2010). Prolactinomas. *New England Journal of Medicine*.

[3] Si M. L., Zhu S., Wu H., Lu Z., Wu F., Mo Y. Y. (2007). miR-21-mediated Tumor Growth. *Oncogene*.

[4] Zhu S., Wu H., Wu F., Nie D., Sheng S., Mo Y. Y. (2008). MicroRNA-21 Targets Tumor Suppressor Genes in Invasion and Metastasis. *Cell Research*.

[5] Moore L. M., Zhang W. (2010). Targeting miR-21 in Glioma: a Small RNA with Big Potential. *Expert Opinion on Therapeutic Targets*.

[6] Medina P. P., Nolde M., Slack F. J. (2010). OncomiR Addiction in an In Vivo Model of microRNA-21- Induced Pre-B-cell Lymphoma. *Nature*.

[7] Giordo R., Ahmadi F. A. M., Husaini N. A. (2024). microRNA 21 and Long Non-coding RNAs Interplays Underlie Cancer Pathophysiology: A Narrative Review. *Non-coding RNA Research*.

[8] Xiong Y., Tang Y., Fan F. (2020). Exosomal Hsa-miR-21-5p Derived from Growth Hormone-Secreting Pituitary Adenoma Promotes Abnormal Bone Formation in Acromegaly. *Translational Research*.

[9] Cui M., Zhang M., Liu H. F., Wang J. P. (2017). Effects of microRNA-21 Targeting PITX2 on Proliferation and Apoptosis of Pituitary Tumor Cells. *European Review for Medical and Pharmacological Sciences*.

[10] Vivanco I., Sawyers C. L. (2002). The Phosphatidylinositol 3-Kinase AKT Pathway in Human Cancer. *Nature Reviews Cancer*.

[11] Gorvin C. M. (2015). The Prolactin Receptor: Diverse and Emerging Roles in Pathophysiology. *Journal of Clinical & Translational Endocrinology Clin. Transl. Endocrinol.*.

[12] Aydin B., Arslan S., Bayraklı F., Karademir B., Arga K. Y. (2022). MicroRNA-Mediated Drug Repurposing Unveiled Potential Candidate Drugs for Prolactinoma Treatment. *Neuroendocrinology*.

[13] The Cancer Genome Atlas Network (2012). Comprehensive Molecular Portraits of Human Breast Tumours. *Nature*.

[14] Liu P., Cheng H., Santiago S. (2011). Oncogenic PIK3CA-driven Mammary Tumors Frequently Recur via PI3K Pathway-dependent and PI3K Pathway-independent Mechanisms. *Nature Medicine*.

[15] Fruman D. A., Rommel C. (2014). PI3K and Cancer: Lessons, Challenges and Opportunities. *Nature Reviews Drug Discovery*.

[16] Xu Y., Sui L., Qiu B., Yin X., Liu J., Zhang X. (2019). ANXA4 Promotes Trophoblast Invasion via the PI3K/Akt/eNOS Pathway in Preeclampsia. *American Journal of Physiology: Cell Physiology*.

[17] Ahmad A., Biersack B., Li Y. (2013). Targeted Regulation of PI3K/Akt/mTOR/NF-Κb Signaling by Indole Compounds and Their Derivatives: Mechanistic Details and Biological Implications for Cancer Therapy. *Anti-Cancer Agents in Medicinal Chemistry*.

[18] Ghoneum A., Said N. (2019). PI3K-AKT-mTOR and NF*κ*B Pathways in Ovarian Cancer: Implications for Targeted Therapeutics. *Cancers*.

[19] Dan H. C., Cooper M. J., Cogswell P. C., Duncan J. A., Ting J. P., Baldwin A. S. (2008). Akt-dependent Regulation of NF-Κb Is Controlled by mTOR and Raptor in Association with IKK. *Genes & Development*.

[20] Du J., Qian J., Zheng B., Xu G., Chen H., Chen C. (2021). miR-21-5p Is a Biomarker for Predicting Prognosis of Lung Adenocarcinoma by Regulating PIK3R1 Expression. *International Journal of General Medicine*.

[21] Chinese Pituitary Tumor Collaborative Group (2014). Chinese Consensus on Diagnosis and Treatment of Pituitary Prolactinoma. *Chinese Medical Journal*.

[22] Levy A. (2004). Pituitary Disease: Presentation, Diagnosis, and Manage-Ment. *Journal of Neurology, Neurosurgery & Psychiatry*.

[23] Andereggen L., Mono M. L., Kellner-Weldon F., Christ E. (2017). Cluster Headache and Macroprolactinoma: Case Report of a Rare, but Potential Important Causality. *Journal of Clinical Neuroscience*.

[24] Oh M. C., Aghi M. K. (2011). Dopamine Agonist-Resistant Prolactino-Mas. *Journal of Neurosurgery*.

[25] Zamanipoor Najafabadi A. H., Zandbergen I. M., de Vries F. (2020). Surgery as a Viable Alternative First-Line Treatment for Prolactinoma Patients. A Systematic Review and Meta-Analysis. *Journal of Clinical Endocrinology and Metabolism*.

[26] Ogiwara T., Horiuchi T., Nagm A., Goto T., Hongo K. (2017). Significance of Surgical Management for Cystic Prolactinoma. *Pituitary*.

[27] Donoho D. A., Laws E. R. (2019). The Role of Surgery in the Management of Prolactinomas. *Neurosurgery Clinics of North America*.

[28] Vasilev V., Daly A. F., Vroonen L., Zacharieva S., Beckers A. (2011). Resistant Prolactinomas. *Journal of Endocrinological Investigation*.

[29] Casanueva F. F., Molitch M. E., Schlechte J. A. (2006). Guidelines of the Pituitary Society for the Diagnosis and Management of Prolactinomas. *Clinical Endocrinology*.

[30] Li Q., Li B., Li Q. (2018). Exosomal miR-21-5p Derived from Gastric Cancer Promotes Peritoneal Metastasis via Mesothelial-To-Mesenchymal Transition. *Cell Death & Disease*.

[31] Ren W., Hou J., Yang C. (2019). Extracellular Vesicles Secreted by Hypoxia Pre-challenged Mesenchymal Stem Cells Promote Non-small Cell Lung Cancer Cell Growth and Mobility as Well as Macrophage M2 Polarization via miR-21-5p Delivery. *Journal of Experimental & Clinical Cancer Research*.

[32] He J. H., Li Y. G., Han Z. P. (2018). The CircRNA-ACAP2/Hsa-miR-21-5p/Tiam1 Regulatory Feedback Circuit Affects the Proliferation, Migration, and Invasion of Colon Cancer SW480 Cells. *Cellular Physiology and Biochemistry*.

[33] Zottel A., Šamec N., Kump A. (2020). Analysis of miR-9-5p, miR-124-3p, miR-21-5p, miR-138-5p, and miR-1-3p in Glioblastoma Cell Lines and Extracellular Vesicles. *International Journal of Molecular Sciences*.

[34] Yang Z., Liao B., Xiang X., Ke S. (2020). miR-21-5p Promotes Cell Proliferation and G1/S Transition in Melanoma by Targeting CDKN2C. *FEBS Open Bio*.

[35] Tang J., Li X., Cheng T., Wu J. (2021). miR-21-5p/SMAD7 axis Promotes the Progress of Lung Cancer. *Thoracic Cancer*.

[36] Zhang L., Yu L., Liu Y., Wang S., Hou Z., Zhou J. (2020). miR-21-5p Promotes Cell Proliferation by Targeting BCL11B in Thp-1 Cells. *Oncology Letters*.

[37] Li G., Yang Y., Xu S., He M., Zhang Z. (2021). mir-21-5p Inhibits the Progression of Human Chondrosarcoma by Regulating CCR7/STAT3/NF-Κb Pathway. *Connective Tissue Research*.

[38] Amaral F. C., Torres N., Saggioro F. (2009). MicroRNAs Differentially Expressed in ACTH-Secreting Pituitary Tumors. *Journal of Clinical Endocrinology and Metabolism*.

[39] Grabowski J. P., Glajzer J., Richter R. (2021). Lymphovascular Space Invasion and Ki67 as Predictors of Lymph Node Metastasis in Primary Low Grade Serous Ovarian Cancer. *International Journal of Gynecological Cancer*.

[40] Sadeghipour A., Mahouzi L., Salem M. M. (2017). Ki67 Labeling Correlated with Invasion but Not with Recurrence. *Applied Immunohistochemistry & Molecular Morphology*.

[41] Roof A. K., Jirawatnotai S., Trudeau T (2018). The Balance of PI3K and ERK Signaling Is Dysregulated in Prolactinoma and Modulated by Dopamine. *Endocrinology*.

[42] Vázquez-Borrego M. C., Fuentes-Fayos A., Herrera-Martínez A (2020). Statins Directly Regulate Pituitary Cell Function and Exert Antitumor Effects in Pituitary Tumors. *Neuroendocrinology*.

[43] The Cancer Genome Atlas Network (2012). Comprehensive Molecular Portraits of Human Breast Tumours. *Nature*.

[44] Liu P., Cheng H., Santiago S (2011). Oncogenic PIK3CA-driven Mammary Tumors Frequently Recur via PI3K Pathway-dependent and PI3K Pathway-independent Mechanisms. *Nature Medicine*.

[45] Cheung L. W. T., Walkiewicz K. W., Besong T. M (2015). Regulation of the PI3K Pathway through a P85*α* Monomer-Homodimer Equilibrium. *Elife*.

[46] Heinrich F., Chakravarthy S., Nanda H. (2015). The PTEN Tumor Suppressor Forms Homodimers in Solution. *Structure*.

[47] Papa A., Pandolfi P. P. (2019). The PTEN^‐^PI3K Axis in Cancer. *Biomolecules*.

